# Treacher Collins syndrome: A case report and review of literature

**DOI:** 10.1002/ccr3.6782

**Published:** 2022-12-27

**Authors:** Nadia Kolsi, Fatma Boudaya, Afef Ben Thabet, Manel Charfi, Chiraz Regaieg, Amira Bouraoui, Ridha Regaieg, Nedia Hentati, Amel Ben Hamed, Abdellatif Gargouri

**Affiliations:** ^1^ Neonatology department Hedi Chaker hospital Sfax Tunisia

**Keywords:** craniofacial dysmorphism, genetic syndrome, multi‐disciplinary medical care, prenatal diagnosis

## Abstract

Treacher Collins syndrome (TCS) is one of the rare genetic syndromes which is specified by symmetrical craniofacial dysmorphism without growth abnormalities or neurological disorders. The inheritance is usually autosomal dominant but sometimes it is a sporadic mutation. Prenatal diagnosis could be realized by genetic testing of a chorionic villus sample or amniocentesis if one of the parents is affected. At birth, the most common features are downward‐sloping palpebral clefts, small badly hemmed and folded ears, and mandibular hypoplasia which could lead to respiratory distress. All of these clinical features exist in our case. Goldenhar syndrome shares with TCS some facial features which are not symmetrical and it is also associated with vertebral abnormalities. Some patients with TCS are exposed to many complications and they require multi‐disciplinary medical care. But all of them need psychiatric care to fight social rejection. The aim of our report is to describe the most common features of TCS and similar syndromes. Also, report the involved genetic mutations, some associated complications, and their management.

## Abstract 2

Faced with facial dysmorphism, the clinician should describe clearly the features in order to be oriented to the diagnosis. Some explorations in particular the genetic study and specialized clinical examination are necessary to: confirm diagnosis, reveal complications, and make easier its management and follow‐up.

## 1 INTRODUCTION

Treacher collins syndrome also named mandibulofacial dysostosis is one of the rare genetic syndromes that causes mostly craniofacial dysmorphism. It was described often as an autosomal dominant disorder with a variable degree of phenotypic expression.^1^ The majority of mutations are discovered in the TCOF1, POLR1C, and POLR1D genes. But, over 50% of cases are sporadic mutations, despite some familial cases which are well known.^2^ The majority of pathology affects structures arising from the first and second pharyngeal arches. Clinical manifestations are usually symmetrical with mandibular, eye, and ears abnormalities. However, patients have no associated developmental delay or neurologic disease.

## 2 CASE REPORT

**FIGURE 1:**
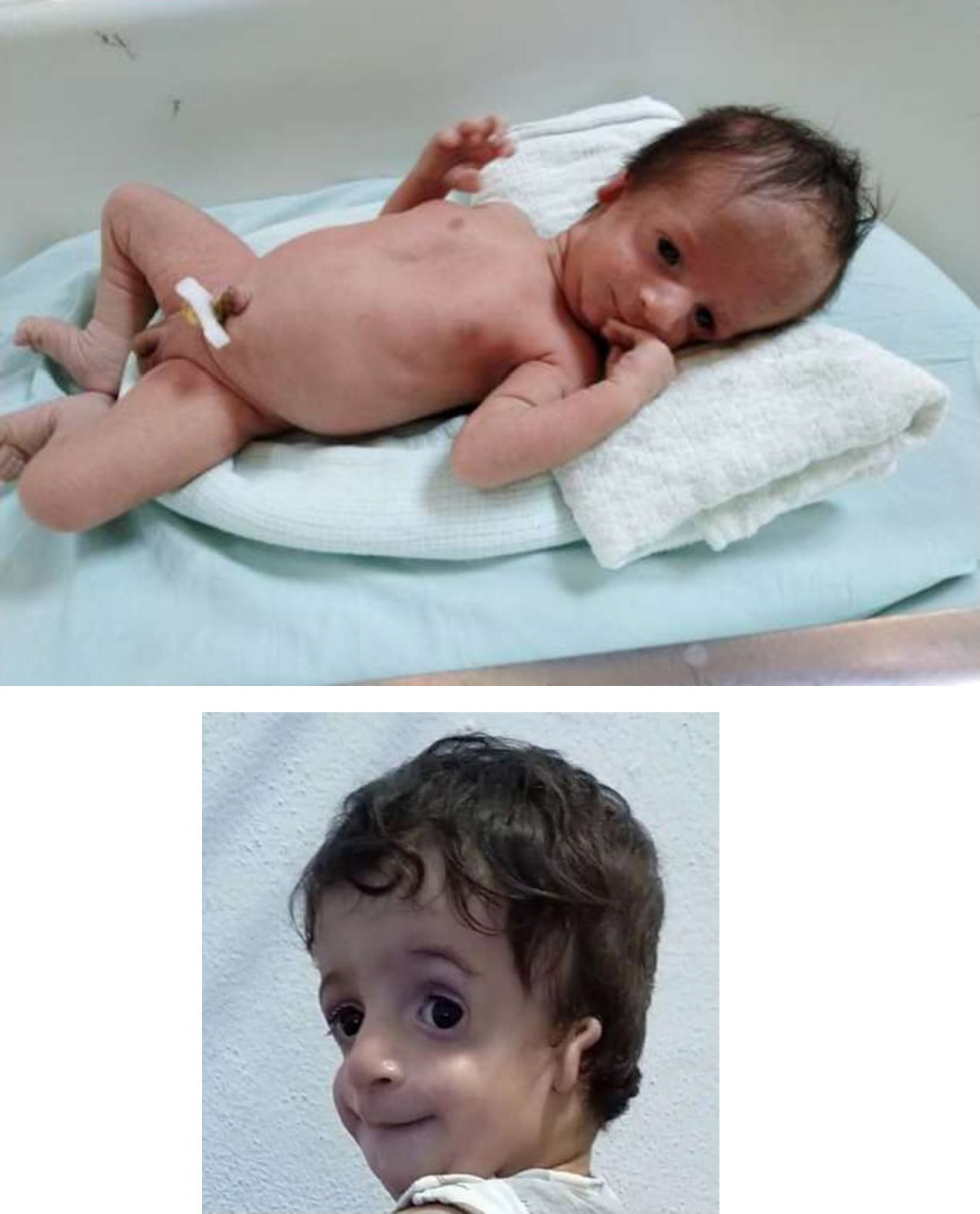
Our patient facial dysmorphia at birth and the age of 8 months

A male newborn whose parents are non‐consanguineous. The mother was 27 years old. She is smoking and addicted to cannabis. The pregnancy was poorly monitored. She was cesarized at 37th weeks of amenorrhea for suspected retroplacental hematoma. At birth, the newborn had respiratory distress requiring oxygenotherapy for only 2 days. The examination found hypotrophy in the 10th percentile and facial dysmorphia. He had a triangular face, receding chin, micrognathia, sunken cheekbones, downward slanting palpebral fissures, absent eyelashes of the lower eyelid, the nose large and beak‐like, microtia with badly hemmed ears folded and auditory external canal stenosis. (Figure 1).

**FIGURE 2:**
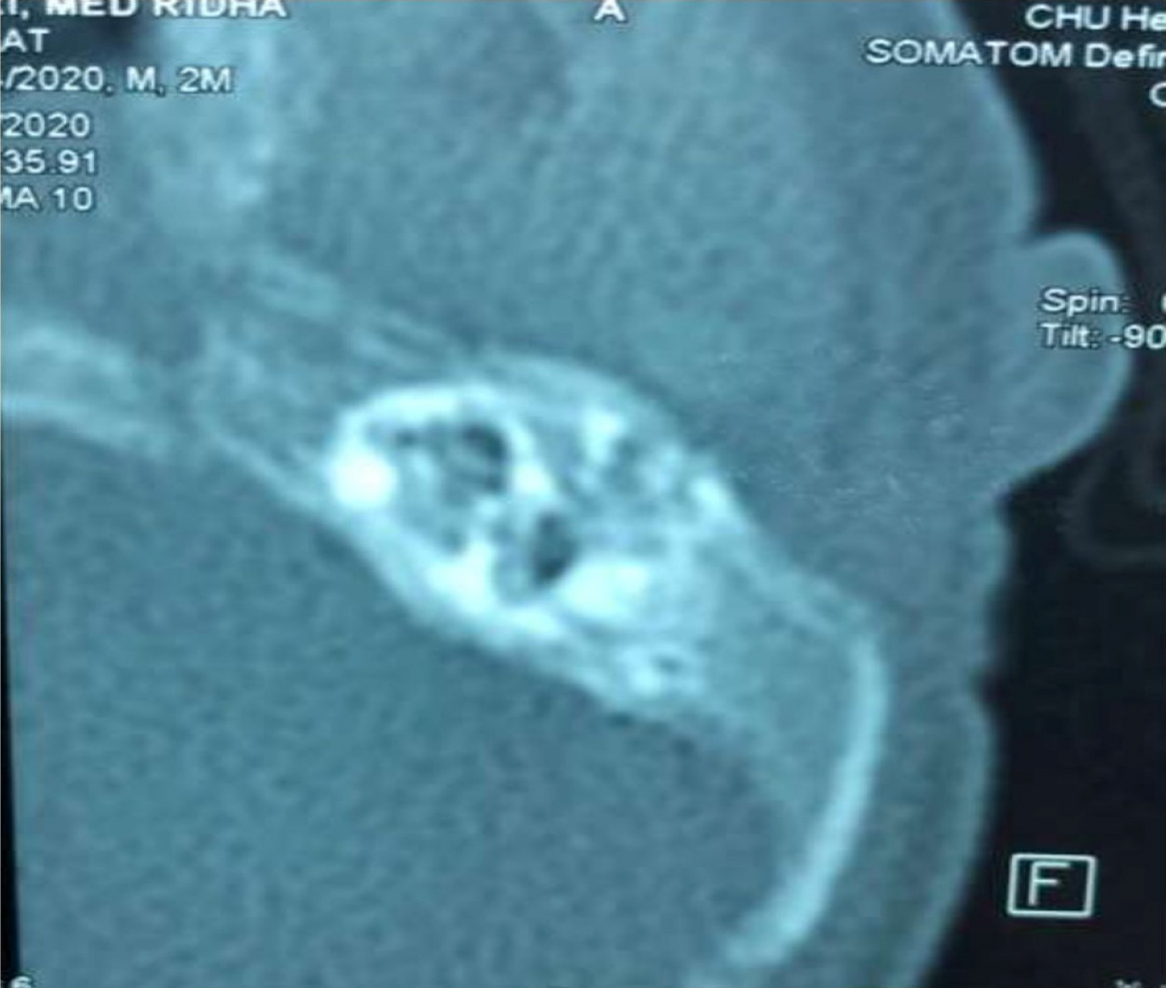
Agenesis of the zygomatic process of the left temporal bone, agenesis of the two external auditory canals, and hypoplasia of the cavities of the two middle ears

Whereas, there are no skeletal anomalies, choanal atresia, cleft palate, or cryptorchidism. A specialized ear, nose, and throat (ENT) examination found a cracked tongue and an unseen uvula. Also, there is only an anthelix, a tragus, and a closed auditory external canal. The ophthalmologist described a telecanthus, good palpebral statics, normal oculomotricity, clear cornea, a normal iris, and a round pupil. The back of the eyes was normal. The scan showed diffuse enlargement of the spaces under the arachnoid, concave mandibular branches, and agenesis of the zygomatic process of the left temporal bone. He had also agenesis of the two external auditory canals and hypoplasia of the cavities of the two middle ears (Figure 2). The genetic study was requested but the result is not yet ready.

## 3 DISCUSSION

The estimated incidence of TCS is 1/50000. There is no predominance of gender or race.^3^ The clinical features vary across affected people and even within members of the same family.^2^ The mode of inheritance is frequently autosomal dominant and rarely autosomal recessive but more than 60% are sporadic and are a result of de novo mutation.^2^, ^4^ The mutated gene responsible is linked to the locus of chromosome 5q32.^5^ Over 120 mutations have been found.^1^ TCOF1, POLR1C, and POLR1D gene mutations are the most commonly implicated.^2^ Recently, Muhammad.k and Omayma. Al have identified a de novo start‐codon loss (c.3G > T) in the EFTUD2 gene in a 4.5‐year‐old female patient with TCS symptoms^17^. Unfortunately, we encountered difficulties in the genetic study of our patient.

Meizner described a fetus in which prenatal sonography showed polyhydramnios, micrognathia, a low‐set ear, and a large cleft lip and palate. The biometric measurements were appropriate for gestational age.^6^ Cohen observed polyhydramnios and growth restriction in a fetus that also had micrognathia and a slanted forehead.^7^ Antimongoloid inclined palpebral fissures had been observed also by Hiroshi Ochi.^8^ While our patient's prenatal ultrasounds showed only growth delay. Also, it is possible to do prenatal genetic testing if one of the parents carries the pathogen.^2^ It requires the same sample of blood from family members and a sample from an amniocentesis or chorionic villus sample. It is usually done in children whose one of the family is affected.^3^ Downward‐sloping palpebral clefts have been described all over the world. The absence of lashes particularly in the medial third of the lower lid is pathognomonic.^1^ Vision loss, strabismus, congenital cataracts, and even occasional microphthalmia or anophthalmia were described.^1^, ^9^ TCS patients usually present with a bilateral deformed ear as microtia or anotia with varying severity. It is associated frequently with external auditory stenosis or atresia which causes conductive hearing loss with a variable degree of deficit. However, the inner ear has often a normal morphology.^1^ A lot of TCS patients (46%) presented respiratory distress from obstructive sleep apnea caused by malar and mandibular hypoplasia. This one is the most common facial feature in TCS patients.^10^, ^11^ The mandibular angle can be poorly developed or may be totally absent. As a result, the decreased height of the lower third of the face is well‐marked.^12^ In addition, the temporal mandibular joint could be deficient and lead to dysfunction and ankylosis.^1^ Other abnormalities were described: choanal atresia, cleft palate, absent parotid glands, cryptorchidism, extremity malformation, renal anomalies, and heart disease. However, these features are not frequent and consistent in affected people. Also, the nose is often described as beaked but many anthropometric studies showed that measurements are normal and the surrounding atrophied tissue is responsible for this appearance.^13^ (Table 1).

**TABLE 1:** Clinical features of the patient in this study, compared to findings in all reported cases

Features		This study	All reported cases^a^
Cranial facial	Micrognthia	Yes	94/96
	Small or dysplastic pinna (e)	No	91/94
	Malar hypoplasia	Yes	85/91
	Hearing loss	Yes	76/90
	Auditory atresia/stenosis	Yes	51/79
	Vestibular system abnormalities^b^	No	16/28
	Ossicular abnormalities^b^	Yes	11/19
	Microphtalmia	No	10/32
	Facial asymmetry	No	30/51
	Preauricular tag (s)	Yes	46/91
	Cleft palate	No	46/95
	Choanal atresia	No	27/89
	Neonatal resuscitation	Yes	14/48
	Tracheostomy	No	11/54
	Limitaion of mouth opening	No	10/87
Extracranial	Thumb anomalies	No	25/83
	Heart defect	No	29/95
	Esophageal atresia	No	24/91
	Renal malformation	No	9/87
Development	Developmental delay	Yes	90/90
	Microcephaly	No	85/96
	Congenital	No	37/58
	Posnatal	Yes	21/57
	Epileptic seizures	No	23/83

^a^ Obtained from Demi et al. (2015: PMID:26118977), Huang et al(2016: PMID26507355), Yu et al.(2018: PMID: 29381487), Narumi‐Kishimoto et al. (2020: PMId32541334), Jacob et al (2020: PMID:32943010), Muhammed KOHAILAN, Omayma Al –Saei (2022: PMID: 35732499) and this study.

^b^ These features were not reported in several studies.

Goldenhar syndrome or vertebral oculo‐auricular dysplasia shares some craniofacial abnormalities with TCS.^10^ However, patients with Goldenhar syndrome are characterized by asymmetrical facial anomalies, vertebral anomalies, and epibulbar dermoids.^5^, ^14^ Nager syndrome shares some facial features and similarities with TCS. But, there are other additional characteristics such as ectropion or out turning of the lower lids. In addition, patients with Nager syndrome have limb anomalies. In fact, thumbs may be hypoplastic, aplastic or duplicated, and the radius and ulna may be fused.^15^

**FIGURE 3:**
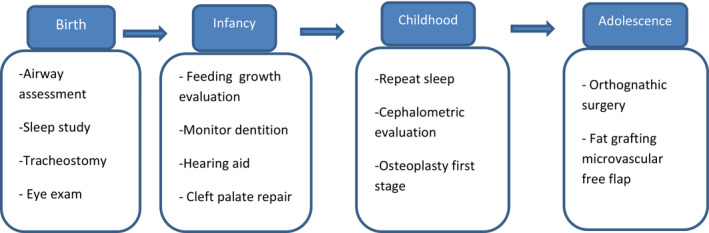
Surgical treatment timeline and staging throughout TCS patient childhood

At birth, some patients with TCS presented respiratory distress because of choanal stenosis or atresia, retropositionned tongue, and obstruction of hypophryngeal spaces by mandibular hypoplasia. Others have suffered from obstructive sleep apnea.^3^, ^9^ Patients with TCS must have audiological testing and early hearing evaluation by a specialist. To acquire communication skills, it is necessary to start speech therapy, bone conduction amplification, and hearing aids before the age of 3. Unfortunately, external ear and auditory canal reconstruction rarely improve hearing.^3^, ^9^ Around 5 and 7 years old, zygomatic and orbital reconstructions are often done.^2^ But, Skeletal remodeling procedures have a high risk of morbidity and mortality. As an alternative, other aesthetic options may help to partially cover retrognathia and midface hypoplasia.^16^ Patients with TCS need a lot of self‐confidence and satisfaction with their appearance to integrate into social life. That is why early psychiatric care is important.^9^ Christopher C. C proposed in his study a surgical treatment timeline and stagging throughout TCS patients’ childhood^1^ (Figure 3).

Actually, our patient suffered from developmental delay and hearing loss which required hearing aids. His parents are accepting his dysmorphism as long as he had no functional complaints.

## 4 CONCLUSION

Treacher Collins syndrome is rare and characterized by symmetrical facial anomalies unlike other craniofacial syndromes. It could be evoked in the preterm stage with ultrasound imaging. It did not associate developmental delay or neurological impairment. Patients with their appearance are faced with problems in integration into social life. Taking care of these patients requires a multidisciplinary team.

## AUTHOR CONTRIBUTIONS

**Nadia Kolsi:** Conceptualization. **Fatma Boudaya:** Writing – original draft. **Afef Ben Thabet:** Visualization. **Manel Charfi:** Resources. **chiraz Regaieg:** Investigation. **Amira Bouraoui:** Validation. **Ridha Regaieg:** Supervision. **nedia Hentati:** Supervision. **Amel Ben Hamad:** Writing – review and editing. **Abdellatif Gargouri:** Supervision.

## CONFLICT OF INTEREST

No conflict of interest in this abstract

## ETHICAL APPROVAL

Yes.

## CONSENT

"Consent was obtained from the parent of the patient to publish this report in accordance with the journal's patient consent policy”

## Data Availability

All data are available.
